# Differential Effects of the Prolyl-Hydroxylase Inhibitor on the Cellular Response to Radiation

**DOI:** 10.3390/ijms26062742

**Published:** 2025-03-18

**Authors:** Masaki Murao, Takahiro Fukazawa, Ujjal K. Bhawal, Nitesh Tewari, Nobuaki Shime, Nobuyuki Hirohashi, Keiji Tanimoto

**Affiliations:** 1 Department of Radiation Disaster Medicine, Research Institute for Radiation Biology and Medicine, Hiroshima University, Hiroshima 734-8553, Japan; murao@hiroshima-u.ac.jp (M.M.); hirohasi@hiroshima-u.ac.jp (N.H.); 2 Radiation Disaster Medicine Support Center, Hiroshima University, Hiroshima 734-8553, Japan; 3 Department of Emergency and Critical Care Medicine, Graduate School of Biomedical and Health Sciences, Hiroshima University, Hiroshima 734-8551, Japan; nshime@hiroshima-u.ac.jp; 4 Natural Science Center for Basic Research and Development, Hiroshima University, Hiroshima 734-8553, Japan; fukazawa.takahiro.ds@ehime-u.ac.jp; 5 Division of Medical Research Support, Advanced Research Support Center, Ehime University, Ehime 791-0204, Japan; 6 Center for Global Health Research, Saveetha Institute of Medical and Technical Sciences, Saveetha Medical College and Hospitals, Saveetha University, Chennai 600077, India; bhawal.ujjal.kumar@nihon-u.ac.jp; 7 Research Institute of Oral Science, Nihon University School of Dentistry at Matsudo, Chiba 271-8587, Japan; 8 Division of Pediatric and Preventive Dentistry, Centre for Dental Education and Research, All India Institute of Medical Sciences, New Delhi 110029, India; dr.nitesht@gmail.com

**Keywords:** radiation, prolyl-hydroxylase inhibitor, hypoxia, p53, DEC2

## Abstract

The prolyl-hydroxylase inhibitor (PHI), used effectively in several countries for the treatment of renal anemia, activates the multifunctional hypoxia-inducible factors (HIFs). While hypoxic conditions in tumors are known to affect the response to radiation therapy, the effect of PHI on the radiation response of cancer cells has not been determined. Hypoxic pretreatment increased the radiation sensitivity of A549 lung adenocarcinoma cells, whereas hypoxic culture after irradiation decreased the radiation sensitivity of HSC2 oral squamous cell carcinoma cells. Treatment of PC9 lung adenocarcinoma and HSC2 cells with the PHI FG-4592 significantly increased radiation resistance, whereas A549 and TIG3 lung fibroblast cells tended to be sensitized, suggesting cell type-specific differential effects of PHI. Quantitative RT-PCR analyses revealed that the basal and radiation-inducible expressions of *DEC2*, *BAX*, and *BCL2* may be related to PHI-mediated radiation responses. Knock-down experiments showed that silencing of DEC2 sensitized both A549 and PC9 cells under PHI-treated conditions. On the other hand, silencing of p53, which regulates *BAX*/*BCL2*, desensitized A549 cells expressing wild-type p53, but not PC9 cells, with mutant-type p53, to irradiation, regardless of whether PHI was treated or not. Taken together, PHI modifies radiation responses in a cell type-specific manner, possibly through DEC2 signaling.

## 1. Introduction

Radiation therapy exerts its therapeutic effect on cancer cells by inducing DNA damage [1,2]. Despite its efficacy, challenges remain, including the development of resistance mechanisms in cancer cells and the potential for collateral damage to healthy normal tissues [3,4]. Consequently, there is an urgent need to identify adjuvants that can sensitize cancer cells to radiation and thereby improve treatment outcomes.

The efficacy of radiation therapy is significantly influenced by the tumor microenvironment, in particular, by the presence of hypoxia, a condition characterized by insufficient oxygen supply [5,6,7]. Hypoxic conditions induce a cascade of molecular events that affect cellular behavior, including cell proliferation, migration, invasion, survival, cell cycle, and DNA repair capacity, leading to induced chemo-radiation resistance [8,9]. Hypoxia-inducible factors (HIFs) play a role in orchestrating the cellular responses to both hypoxia and radiation [5,6,7]. In our previous studies, we elucidated the molecular mechanisms underlying the complex relationship between hypoxia and radiation response and demonstrated the role of the HIF-DEC transcription factor axis in the DNA damage response system, including damage recognition, repair, and apoptosis [10,11]. Our comprehensive gene expression analysis revealed systemic suppression of DNA damage response-related gene expression in cancer cells under hypoxic conditions by DEC transcription factors, resulting in inhibition of radiation-induced DNA damage responses. A better understanding of the detailed mechanistic basis of how hypoxia modulates the radiation response is of paramount importance to optimize therapeutic strategies for cancer treatment.

In recent years, prolyl-hydroxylase inhibitors (PHIs) have been used to treat renal anemia in several countries [12,13,14]. Prolyl hydroxylase domain proteins (PHDs) are central to the regulation of hypoxia-inducible factors (HIFs) and serve as cellular oxygen sensors [14,15]. Under normoxic conditions, PHDs hydroxylate specific proline residues on the HIF-α subunit, marking it for recognition by the von Hippel-Lindau (VHL) E3 ubiquitin ligase complex. This process leads to ubiquitination and subsequent proteasomal degradation of HIF-α, thereby preventing the activation of hypoxia-responsive genes. Under hypoxic conditions, the activity of PHDs diminishes due to reduced oxygen availability, resulting in the stabilization and accumulation of HIF-α. Stabilized HIF-α translocates to the nucleus, dimerizes with HIF-β, and activates the transcription of genes involved in angiogenesis, erythropoiesis, and metabolism, facilitating cellular adaptation to low oxygen environments [14,15]. PHIs typically function by chelating the iron ions essential for PHD enzymatic activity or by competitively binding to the active site of the enzyme. These inhibitors, originally recognized for their effects on stabilizing the HIF protein as described above, have attracted attention for their diverse effects on cellular processes including in hypoxic environments. Whether prolyl-hydroxylase inhibition influences the cellular response to radiation therapy has not been determined.

Several PHIs have been developed and approved for clinical use, and roxadustat (FG-4592) was the first HIF-PHD inhibitor to be approved for oral administration, providing a convenient alternative to traditional injectable erythropoiesis-stimulating agents [16]. In this study, we, therefore, investigated the complex interplay between prolyl-hydroxylase inhibition (FG-4592) and radiation therapy, intending to explore its potential as an adjuvant therapeutic strategy for cancer treatment.

## 2. Results

### 2.1. Effects of Hypoxia on Radiation-Induced Cell Growth Inhibition

**Figure 1:**
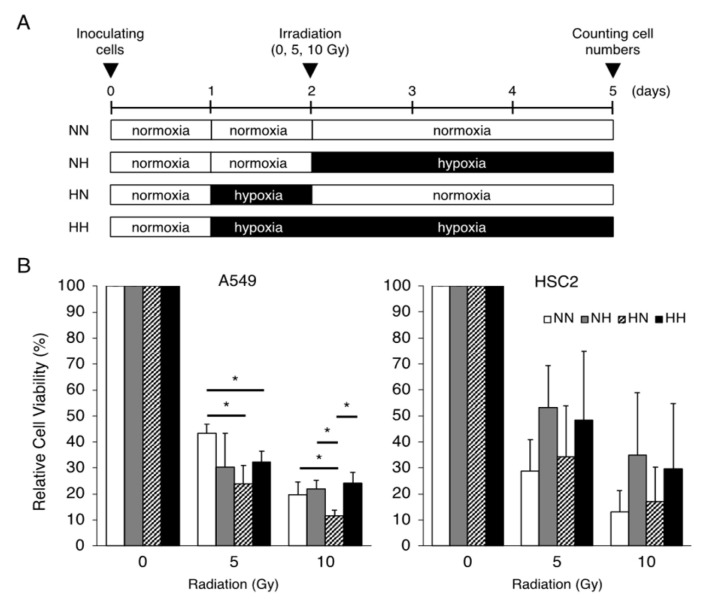
Effects of the timing of hypoxic treatments on radiation-induced cell growth inhibition. (**A**) Schematic of the experimental analysis. Cells were cultured under normoxic (21% O_2_) or hypoxic (1% O_2_) conditions, as indicated. All cells were irradiated under normoxic conditions. (**B**) Relative cell viability of A549 and HSC2 cells after γ-irradiation irradiation (0, 5, or 10 Gy) in the treatment groups was evaluated by MTT assays. Data are presented as mean and SD (*n* = 3); * *p* < 0.05 (for comparison with NN).

To clarify the differences in the hypoxic effects of radiation exposure due to differences in the timing of hypoxic stimulation, the sensitivity of cancer cell lines to γ-irradiation under various combinations of normoxic and hypoxic conditions was examined (Figure 1A). Hypoxic pretreatment of A549 lung adenocarcinoma cells sensitized the cells to irradiation; hypoxic treatment of HSC2 head and neck cancer cells after irradiation tended to increase cell viability, but without significance (Figure 1B).

### 2.2. Effect of PHI on HIF-1α Protein and Target Gene Expressions

**Figure 2:**
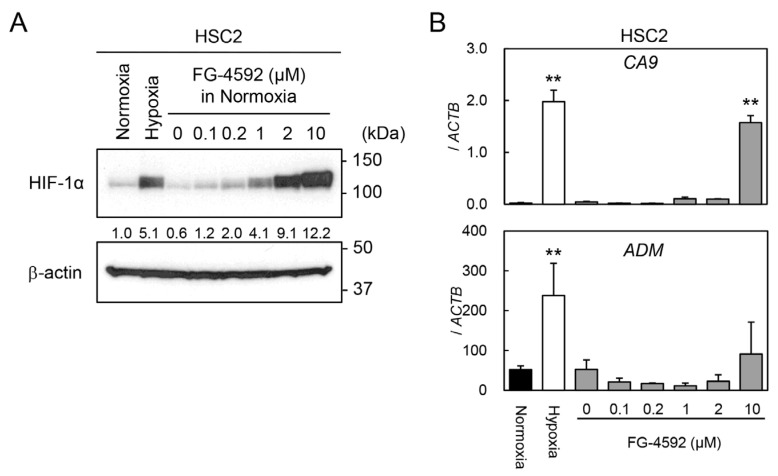
Induction of HIF-1α protein and its target gene expression by FG-4592. (**A**) Protein levels of HIF-1α in HSC2 cells treated with normoxia, hypoxia, or various concentrations of FG-4592 were analyzed by immunoblotting. β-actin was used as an internal loading control. Relative expression levels of HIF-1α protein were calculated with β-actin expression as the denominator for each sample, and the values are shown between panels. (**B**) Expression levels of *CA9* and *ADM* in HSC2 cells treated with normoxia (black bar), hypoxia (white bar), or different concentrations of FG-4592 (gray bars) were determined by quantitative RT-PCR. Relative gene expression levels were calculated using *ACTB*. Data are presented as mean and SD (*n* = 3); ** *p* < 0.01 (for comparison with Normoxia).

To optimize the concentration of the PHI FG-4592 for experiments, we preliminarily investigated the effects of FG-4592 on HIF-1α protein stabilization and target gene expressions in HSC2 cells by immunoblotting and RT-qPCR analysis. The HIF-1α protein was induced under hypoxic conditions (1% O_2_), whereas it was barely detectable under normoxic conditions (21% O_2_) (Figure 2A). Treatment with FG-4592 for 24 h induced a dose-dependent stabilization of HIF-1α protein, and the concentrations between 2 and 10 µM had similar effects on HIF-1α protein induction under hypoxic conditions. Similar effects of FG-4592 were observed in other cell lines derived from different organs, although there was little variation between cell lines (Figure S1). The expression levels of the HIF-1 target genes *CA9* and *ADM* were induced by 10 µM FG-4592. Therefore, 10 µM FG-4592 was chosen for the following experiments.

### 2.3. Effects of FG-4592 Treatment on Radiation-Induced Cell Growth Inhibition

**Figure 3:**
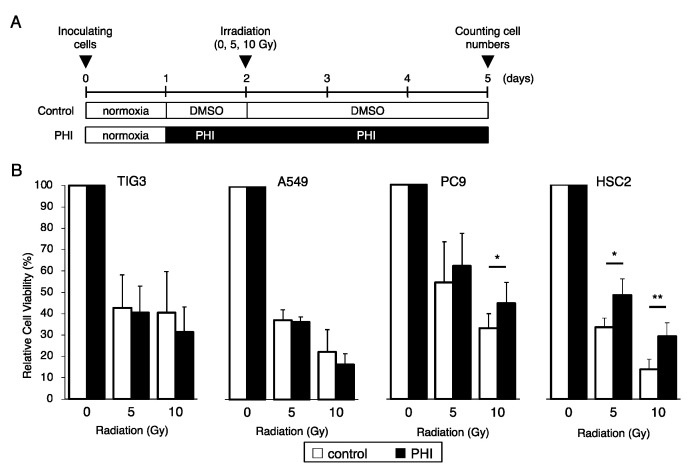
Effects of FG-4592 treatment on radiation-induced cell growth inhibition. (**A**) Schematic of the experimental analysis. Cells were cultured without (control) or with 10 µM of FG-4592 (PHI) for the indicated time periods. All cells were irradiated under normoxic conditions. (**B**) The relative cell viability of TIG3, A549, PC9, and HSC2 cells after γ-irradiation (0, 5, or 10 Gy) under the indicated conditions was evaluated by the MTT assay. Data are presented as mean and SD (*n* = 3); * *p* < 0.05; ** *p* < 0.01 (for comparison with control).

The sensitivity of several cell lines to γ-irradiation under FG-4592-treated conditions is shown in Figure 3A. FG-4592 treatment significantly increased the cell viability of PC9 lung adenocarcinoma cells and HSC2 head and neck cancer cells after γ-irradiation (Figure 3B). On the other hand, PHI treatment of TIG3 lung fibroblast cells and A549 lung adenocarcinoma cells did not seem to affect their irradiation sensitivity. Similar results were also observed with treatment with another PHI, GSK1278863, in HSC2 and A549 cells (Figure S2).

### 2.4. Effects of FG-4592 Treatment on the Expressions of Hypoxia, Apoptosis, and Cell Cycle-Related Genes

**Figure 4:**
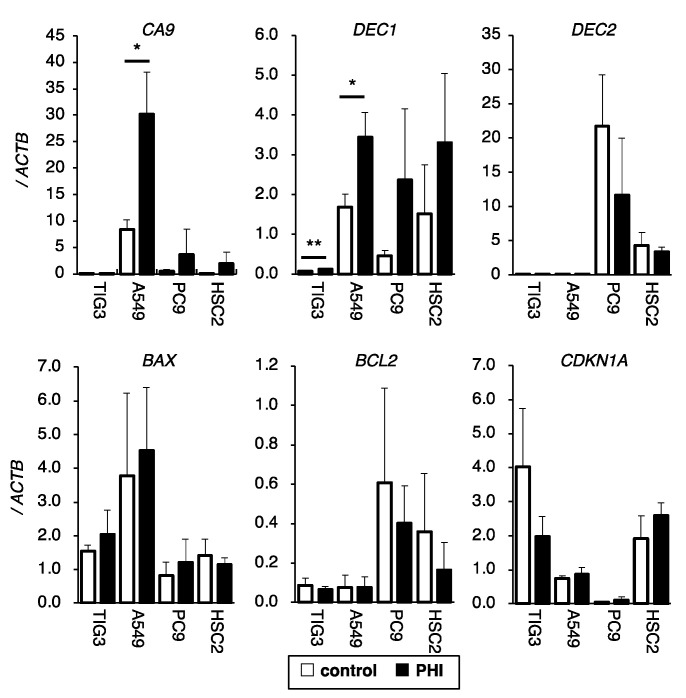
Effects of FG-4592 treatment on the expression of hypoxia, apoptosis, and cell cycle-related genes. The expression of *CA9*, *DEC1*, *DEC2* (hypoxia-inducible genes), *BAX*, *BCL2* (apoptosis-regulating genes), and *CDKN1A* (cell cycle-regulating gene) in FG-4592 (PHI)-treated cells were analyzed by quantitative RT-PCR. The relative gene expression levels were calculated as the ratio relative to that of *ACTB*. Data are presented as mean and SD (*n* = 3); * *p* < 0.05; ** *p* < 0.01 (for comparison with control).

The effects of FG-4592 treatment for 24 h on the expressions of HIF target genes and genes related to apoptosis and cell cycle control were evaluated by RT-qPCR. *CA9* and *DEC1* were induced to different degrees by FG-4592 in different cell lines. *DEC2* was detected in PC9 and HSC2 cells but not in TIG3 and A549 cells, and *DEC2* was not induced by FG-4592 treatments in the tested cell lines under these conditions (Figure 4). The expression of the cell cycle-regulating gene *CDKN1A* varied between cell lines. Except for the increase in *CA9* and *DEC1* in A549 and TIG3 cells, respectively, FG-4592 did not affect the expression of the indicated genes (Figure 4).

### 2.5. Effects of Irradiation on Hypoxia-, Apoptosis-, and Cell Cycle-Related Gene Expressions in FG-4592-Treated Cells

**Figure 5:**
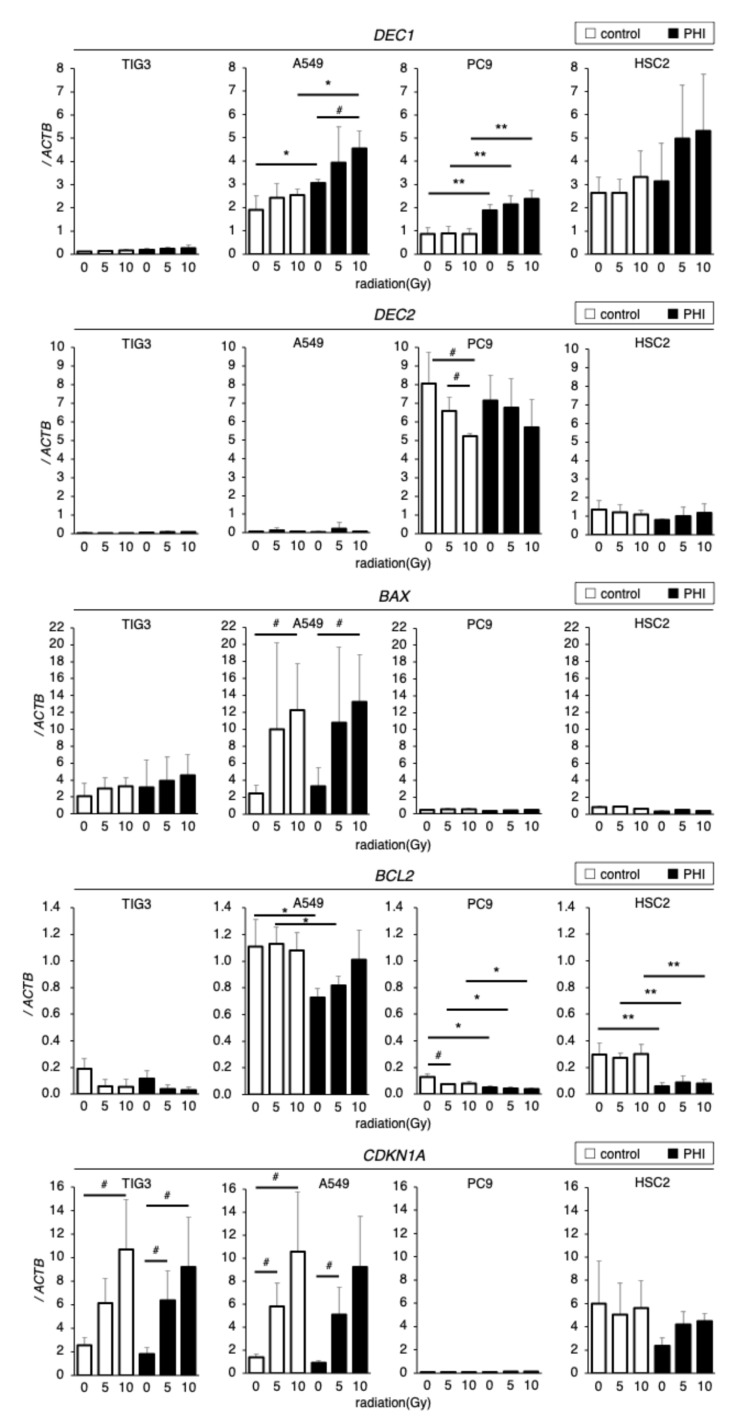
Effects of irradiation on hypoxia, apoptosis, and cell cycle-related gene expressions in FG-4592 (PHI)-treated cells. Expression of *DEC1* and *DEC2* (hypoxia-inducible genes), *BAX, BCL2* (apoptosis-regulating genes), and *CDKN1A* (cell cycle-regulating gene) in FG-4592 (PHI)-treated and/or irradiated cells was analyzed with quantitative RT-PCR. Relative gene expression levels were calculated as the ratio to that of *ACTB*. Data are presented as mean and SD (*n* = 3); * *p* < 0.05; ** *p* < 0.01 (for comparison with control); # *p* < 0.05 (for comparison between experimental samples).

The effects of irradiation on the expressions of HIF target genes and genes related to apoptosis and cell cycle control in FG-4592-treated cells were evaluated by RT-qPCR. *DEC1* was detectable in A549, PC9, and HSC2 cells, whereas it was barely observed in TIG3 cells; FG-4592 treatment significantly increased *DEC1* in A549 and PC9 cells (Figure 5). Irradiation significantly increased *DEC1* expression in A549 cells and slightly in PC9 and HSC2 cells under FG-4592-treated conditions. *DEC2* was detected only in PC9 and HSC2 cells but not in TIG3 and A549 cells. Irradiation significantly decreased *DEC2* expression in PC9 cells compared to control conditions. *DEC2* expression in FG-4592-treated PC9 and control HSC2 cells tended to be decreased by irradiation but without significance. Expression of the pro-apoptotic gene *BAX* was detected only in TIG3 and A549 cells but not in PC9 and HSC2 cells. Irradiation significantly increased *BAX* expression in A549 cells and slightly increased, without significance, in TIG3 cells. *BCL2* expression varied between cell lines. FG-4592 treatment significantly decreased *BCL2* expression in A549, PC9, and HSC2 cells, and irradiation decreased *BCL2* expression in PC9 cells. Expression of the cell cycle-regulating gene *CDKN1A* varied between cell lines. Irradiation significantly increased *CDKN1A* expression in TIG3 and A549 cells, but no effects of FG-4592 treatment were observed. The results seemed to be complicated, but *DEC2* was expressed only in PC9 and HSC2, whose sensitivities to radiation were affected by FG-4592 treatment. p53 target genes, *BAX* and *CDKN1A*, were decreased by radiation only in TIG3 and A549 cells with wild-type p53.

### 2.6. Effects of Gene Knock-Down on Radiation Sensitivity in PHI-Treated Cells

**Figure 6:**
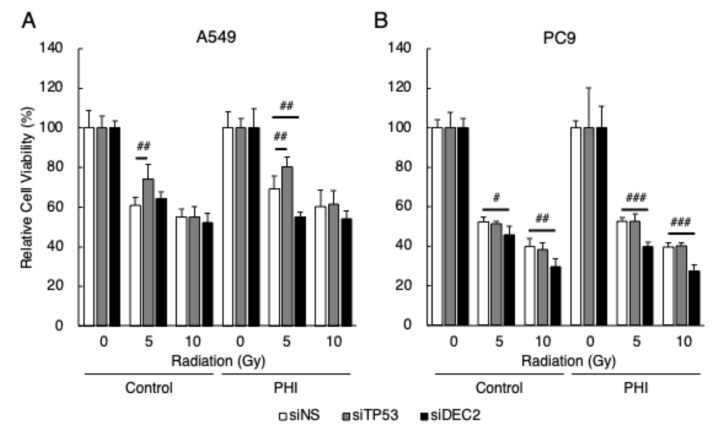
Effects of gene knock-down on radiation sensitivity in FG-4592-treated cells. Cells were transfected with siRNA specific for *TP53* and *DEC2*. The relative cell viability of A549 (**A**) and PC9 (**B**) cells after γ-irradiation (0, 5, or 10 Gy) under FG-4592 (PHI)-treated conditions was evaluated by the MTT assay. Data are presented as mean and SD (*n* = 3); # *p* < 0.05; ## *p* < 0.01, ### *p* < 0.001 (for comparison between experimental samples).

Since the expression of *DEC2* was detected only in the cells whose sensitivity to radiation was decreased by FG-4592 treatment, and the expressions of p53 target genes, *BAX* and *CDKN1A*, were regulated by radiation only in the cells whose sensitivity to radiation was not affected by FG-4592, the effects of *DEC2* or *TP53* knock-down on radiation sensitivity in A549 and PC9 cells with FG-4592 treatment were evaluated. Knock-down effects were evaluated by RT-qPCR (Figure S3). Knock-down of *TP53* significantly decreased the sensitivity to radiation in A549 cells under both control and FG-4592-treated conditions, but had no effect in PC9 cells, as expected (Figure 6). Knock-down of *DEC2* significantly increased the sensitivity to radiation in A549 cells under FG-4592-treated conditions and in PC9 cells under both control and FG-4592-treated conditions.

## 3. Discussion

The interaction between hypoxia and radiation therapy represents a complex landscape in cancer treatment [1,2,3,4]. Hypoxic environments induce a cascade of molecular events that affect cellular behavior, including cell proliferation, migration, invasion, survival, cell cycle, and DNA repair capacity, leading to increased radiation resistance [3,4]. The results presented in this study shed light on the intricate interplay between PHI and radiation therapy and provide insight into potential therapeutic strategies.

We have shown that there are two types of cells: one that acquires radiation resistance under hypoxic conditions and another that does not change. These results underscore the context-dependent nature of hypoxia in modulating radiation responses, which may vary among different cell types. It is important to elucidate the molecular mechanisms of radiation responses through a comparative analysis of these two cell types. We found that a hypoxia mimic reagent, a PHI, FG-4592, affected radiation responses similarly to hypoxia, suggesting an important role of intracellular hypoxic signaling as well as environmental hypoxia. PHIs have been used effectively to treat renal anemia in several countries [12,13,14,15], suggesting their safety in clinical treatments. For the potential application of PHIs with radiation treatment, it is critical to understand the molecular mechanisms of their differential effects on radiation responses. Therefore, we investigated the differential responses to irradiation by analyzing a number of genes. The HIF-1α protein induced by FG-4592 may not be sufficient to induce target gene expression as much as the levels induced by real hypoxia, suggesting the importance of post-translational protein modification in HIF-1α activation under hypoxic conditions [17,18]. Therefore, the induction of the HIF-1α protein and its target genes by FG-4592 suggests the importance of optimizing drug concentrations to achieve the desired therapeutic effects. In this study, experiments with higher concentrations could not be conducted due to the characteristics of the formulation, but, in order to obtain more practical effects, it will be necessary to try reagents with higher concentrations. Although 1% O_2_ condition was used as the hypoxic condition in this study, it will be interesting to compare milder (5–10% O_2_) or severe (<0.5% O_2_) conditions as well as higher doses of FG-4592. In addition, shorter (a few hours to 12 h) or longer (a few weeks) incubations under hypoxic conditions, as well as FG-4592, will also be important to gain additional insight. Methodologically, the gold standard for evaluating the radiation response of cells is the colony formation assay, but, in this study, the MTT assay was used. The colony formation assay is very effective for evaluation over a period of several weeks, but the MTT assay is more advantageous for evaluation over a short period of time, from several hours to several days. In future studies, it will be necessary to use methods suitable for these conditions.

The effects of FG-4592 treatment on radiation-induced cell growth inhibition varied among different cell lines as well as under hypoxic conditions. FG-4592 treatment significantly increased cell viability in lung adenocarcinoma cells (PC9) after irradiation and tended to render head and neck cancer cells (HSC2) resistant to radiation, although not significantly. FG-4592 treatment did not appear to alter the sensitivity of lung fibroblast cells (TIG3) and lung adenocarcinoma cells (A549) to radiation. These differential responses highlight the complexity of cellular signaling pathways and underscore the importance of cancer-specific and/or personalized treatment approaches in cancer therapy. Examination of the gene expression related to hypoxia, apoptosis, and cell cycle control provided additional insight into the molecular mechanisms underlying the observed responses. The differential expression of HIF target genes and apoptosis-related genes in different cell lines suggested different regulatory mechanisms governing cellular responses to FG-4592 treatment and irradiation [19,20,21]. Among these mechanisms, our results suggested the importance of *DEC2* expression in cells whose sensitivity to radiation was altered by FG-4592 treatment. DEC2 (also known as BHLHE41) is a basic helix–loop–helix transcription factor that has been identified as a key regulator in various cellular processes and has been implicated in the transcriptional regulation of hypoxia-related genes [22]. Functional hypoxia response elements (HREs) in the promoter region of *DEC1* and *DEC2* genes were identified, suggesting their role in mediating cellular responses under hypoxic conditions. Subsequent studies further highlighted the role of DEC2 in DNA damage response mechanisms under hypoxic conditions [10,11]: DEC2 is involved in the transcriptional regulation of DNA damage response genes, demonstrating the importance of the HIF-DEC axis in the response to DNA damage under stress conditions. In analogy to these findings, FG-4592 may repress the expression of DNA damage response genes through the activation of HIF-DEC signaling, resulting in acquired radiation resistance in our study. The differential expression of p53 target genes also varied between cell lines. The TIG3 and A549 cells, which harbor functional wild-type p53, expressed pro-apoptotic and cell cycle-regulating genes and responded to irradiation [23,24]. The PC9 and HSC2 cells, in which radiation-induced expression of p53 target genes was lost, expressed the hypoxic transcription factor gene *DEC2*. The results of this study strongly suggest an association between p53 and DEC2, although the regulatory mechanisms remain unclear and require further investigation.

The effects of *DEC2* or *TP53* gene knock-down on radiation sensitivity further elucidate the role of these genes in modulating cellular responses to radiation therapy under FG-4592-treated conditions. Our results showed that *DEC2* knock-down increased radiation sensitivity in both A549 and PC9 cells under FG-4592-treated conditions, whereas *TP53* knock-down decreased the sensitivity to radiation only in A549 cells under both control and FG-4592-treated conditions. These results underscore the intricate interplay of genetic factors in determining cellular responses to radiation therapy. In this study, DEC2 has emerged as an important factor in the response to irradiation under PHI treatments; under such conditions, PHI could repress the expression of DNA damage response genes through the activation of HIF-DEC signaling. Therefore, regulation of DEC2 may lead to the development of a novel therapeutic strategy to control radiation responses.

Prolyl hydroxylase domain (PHD) inhibitors have emerged as an important class of drugs that prevent the degradation of hypoxia-inducible factors (HIFs), thereby increasing erythropoietin (EPO) production and stimulating erythropoiesis. These inhibitors have been widely used in the clinical management of anemia associated with chronic kidney disease (CKD) due to their ability to mimic hypoxic conditions and promote endogenous EPO synthesis [25]. Several PHD inhibitors have been developed and approved for clinical use, including roxadustat (FG-4592), daprodustat (GSK1278863), vadadustat (MT-6548), molidustat (BAY 85-3934), and enarodustat (JTZ-951). While these inhibitors share a common mechanism of action in stabilizing HIFs and promoting erythropoiesis, clinical trials have shown both common and distinct effects among them. Despite shared benefits in CKD patients, there are notable differences among PHD inhibitors in terms of safety profiles, iron metabolism effects, and dosing regimens [26,27]. Adverse effects vary across different drugs, with vadadustat being associated with hypertension and nausea, while daprodustat has been reported to cause abdominal discomfort and complications related to vascular access. In addition, some inhibitors have variable effects on iron metabolism, requiring individualized monitoring and management of iron status during treatment. Taken together, PHIs commonly activate the HIF-DEC axis through PHD inhibition, but each PHI may also have distinct functions, suggesting differential effects on radiation response. In this study, FG-4592 and GSK1278863 showed a similar effect on radiation responses in HSC2 and A549 cells, although it is still a preliminary experiment. Further comparable experiments with other PHIs are desired for better understanding and development of therapeutic strategy.

In conclusion, this study provides valuable insights into the complex relationship between PHI and radiation therapy in cancer treatment. These findings underscore the importance of considering the tumor microenvironment and genetic factors, particularly DEC2, in optimizing therapeutic strategies for specific cancers and individual patients. Further research is needed to elucidate the underlying mechanisms and to translate these findings into clinical applications aimed at improving treatment outcomes in cancer patients.

## 4. Materials and Methods

### 4.1. Chemicals

All chemicals were of analytical grade and were purchased from FUJIFILM Wako Pure Chemicals (Osaka, Japan), Sigma (St. Louis, MO, USA), AdooQ Bioscience (Irvine, CA, USA), or MedChemExpress (Monmouth Junction, NJ, USA).

### 4.2. Cell Culture

Human lung fibroblast (TIG3), lung adenocarcinoma (A549 and PC9), oral squamous cell carcinoma (HSC2), osteosarcoma (Saos2), chondrosarcoma (OUMS27), and hepatoblastoma (HepG2) cell lines were obtained from the Japanese Cancer Research Resource Bank (JCRB) and have been maintained as original stocks. Cells were maintained in Dulbecco’s Modified Eagle Medium (DMEM) or RPMI1640 (NACALAI TESQUE, Inc., Kyoto, Japan) containing 10% fetal bovine serum (FBS; BioWhittaker, Verviers, Belgium) and 100 µg/mL of Kanamycin Sulfate Solution (FUJIFILM Wako Pure Chemical Corporation, Osaka, Japan), and were used within 3 months of passage from the original stocks. Cells were cultured under normoxic (21% O_2_) or hypoxic (1% O_2_) conditions in a hypoxic chamber. To mimic hypoxia, cells were treated with 10 µM of FG-4592 or 10 µM of GSK1278863, which produced an equivalent response to 1% O_2_.

### 4.3. Cell Irradiation

Irradiation was performed with 5 or 10 Gy of 137Cs γ-irradiation in a Gammacell^®^ 40 Exactor (Nordion International, Inc., Nordion, Ottawa, ON, Canada) for 375–750 sec under normoxic conditions.

### 4.4. Cell Proliferation and Drug Sensitivity Analyses

Cell proliferation capacity was evaluated by the MTT assay or by counting the number of cells. Cells (4 × 10^3^) were seeded on a 96-well plate, cultured for 1 day, and then treated with FG-4592 for the indicated time periods. The MTT [3-(4,5-dimethylthial-2-yl)-2,5-diphenyltetrazalium bromide] formazan precipitate was dissolved in DMSO, and the absorbance at 570 and 650 nm (reference) was measured using an EMax^®®^ Endopoint ELISA Microplate Reader (Molecular Devices Corp., Sunnyvale, CA, USA).

Cell numbers were counted using an INCell Analyzer 2000 (GE Healthcare Japan, Tokyo, Japan) after DAPI staining. All experiments were repeated at least three times under similar conditions.

### 4.5. Immunoblotting Analysis

Whole-cell extracts were prepared from cultured cells, as described previously [28]. Fifty µg of extracts were separated by SDS-polyacrylamide gel electrophoresis and transferred to PVDF membranes. Immunoblotting was performed according to standard procedures. Anti-HIF-1α (#3094, Cell Signaling TECHNOLOGY, Danvers, MA, USA) and anti-β-actin (A5441, Sigma) were used as primary antibodies, diluted at 1:500. Anti-rabbit IgG or anti-mouse IgG horseradish peroxidase conjugate (#7076, #7074, CST) was used as a secondary antibody, diluted at 1:4000. Bands were visualized with the enhanced chemiluminescence reagent SuperSignal West Pico PLUS (Thermo Fisher Scientific K.K., Tokyo, Japan).

### 4.6. Quantitative Reverse Transcription-Polymerase Chain Reaction (RT-PCR) Analysis

Total RNA was prepared from frozen cell pellets using NucleoSpin^®®^ RNA (MACHEREY-NAGEL GmbH&Co. KG, Düren, Germany), according to the manufacturer’s instructions. A total of 1 µg of total RNA was reverse-transcribed using a High-Capacity cDNA Archive^TM^ Kit (Applied Biosystems, Foster City, CA, USA). An aliquot of cDNA was subjected to quantitative RT-PCR using primers (200 nM final concentration each) and MGB probe sets (100 nM final concentration; the Universal Probe Library [UPL], Roche Diagnostics, Tokyo, Japan) for *CA9*, *DEC1* (*BHLHE40*), *DEC2* (*BHLHE41*), *BAX*, *BCL2*, *CDKN1A*, and *TP53*. Primer sequences are listed in Table S1. A pre-developed TaqMan Assay Reagent (Applied Biosystems) for *ACTB* (4326315E, Applied Biosystems) was used as an internal control. PCR reactions were performed using a 7500 Real-Time PCR System (Applied Biosystems) under standard conditions. Gene expression levels were standardized using pooled cDNA from 17 non-identical cancer cell lines, and relative expression was determined using *ACTB* expression [29].

### 4.7. Knock-Down Analysis

A549 or PC9 cells were transfected with siRNA specific for *TP53* (siTP53, SI02655170) or *DEC2* (siDEC2, SI00312004) or with non-specific (siNS, No. 1027310) siRNA (QIAGEN, Inc., Valencia, CA, USA) using Lipofectamine^TM^ RNAiMAX (Thermo Fisher Scientific) for 24 h. Cells were harvested and stored at −80 °C until use.

### 4.8. Statistical Analysis

All statistical tests were performed using EZ-R version 1.54. The Tukey–Kramer method or *t*-test was used to determine the *P*-value for post-hoc pairwise comparisons [19] when an ANOVA test revealed significant heterogeneity.

## Data Availability

The original contributions presented in this study are available in the article/Supplementary Materials; for further information, please contact the corresponding author.

## Supplementary Materials

The following supporting information can be downloaded at: https://www.mdpi.com/article/10.3390/ijms26062742/s1.
