# Supplementary material for: Differential Effects of the Prolyl-Hydroxylase Inhibitor on the Cellular Response to Radiation
Source: Int J Mol Sci. 2025 Mar 18;26(6):2742. doi: 10.3390/ijms26062742 (PMC11943049; doi:10.3390/ijms26062742)
Supplement: Supplementary file 1 [file ijms-26-02742-s001.zip › ijms-3455886-supplementary.pdf]

Supplementary Materials

Table S1. Primer and probe sets for RT-qPCR.

CA9-F: 5′-CCTTTGCCAGAGTTGACGAG-3′
CA9-R: 5′-GCAACTGCTCATAGGCACTG-3′
CA9-probe: UPL #25 (Roche)

ADM-F: 5′-GCCTGCCCAGACCCTTAT-3′
ADM-R: 5′-GTAGCGCTTGACTCGGATG-3′
ADM-probe: UPL #57 (Roche)

DEC1-F: 5′-GACTGGAGCACGGAGACCT-3′
DEC1-R: 5′-GGTGCGGCAATTTGTAGG-3′
DEC1-probe: UPL #56 (Roche)

DEC2-F: 5′-CTACTGCGTGCCCGTCAT-3′
DEC2-R: 5′-CGGTGTCCGTGTCGTTCT-3′
DEC2-probe: UPL #26 (Roche)

BCL2-F: 5′-AGTACCTGAACCGGCACCT-3′
BCL2-R: 5′-GCCGTACAGTTCCACAAAGG-3′
BCL2-probe: UPL #75 (Roche)

BAX-F: 5′-CCATCATGGGCTGGACAT-3′
BAX-R: 5′-CACTCCCGCCACAAAGAT-3′
BAX-probe: UPL #69 (Roche)

*CDKN1A*_F: 5′-TCACTGTCTTGTACCCTTGTGC-3′
*CDKN1A*_R: 5′-GGCGTTTGGAGTGGTAGAAA-3′
*CDKN1A*_probe: UPL #32 (Roche)

*TP53*_F: 5′-CCCCAGCCAAAGAAGAAAC-3′
*TP53*_R: 5′-AACATCTCGAAGCGCTCAC-3′
*TP53*_probe: UPL #58 (Roche)

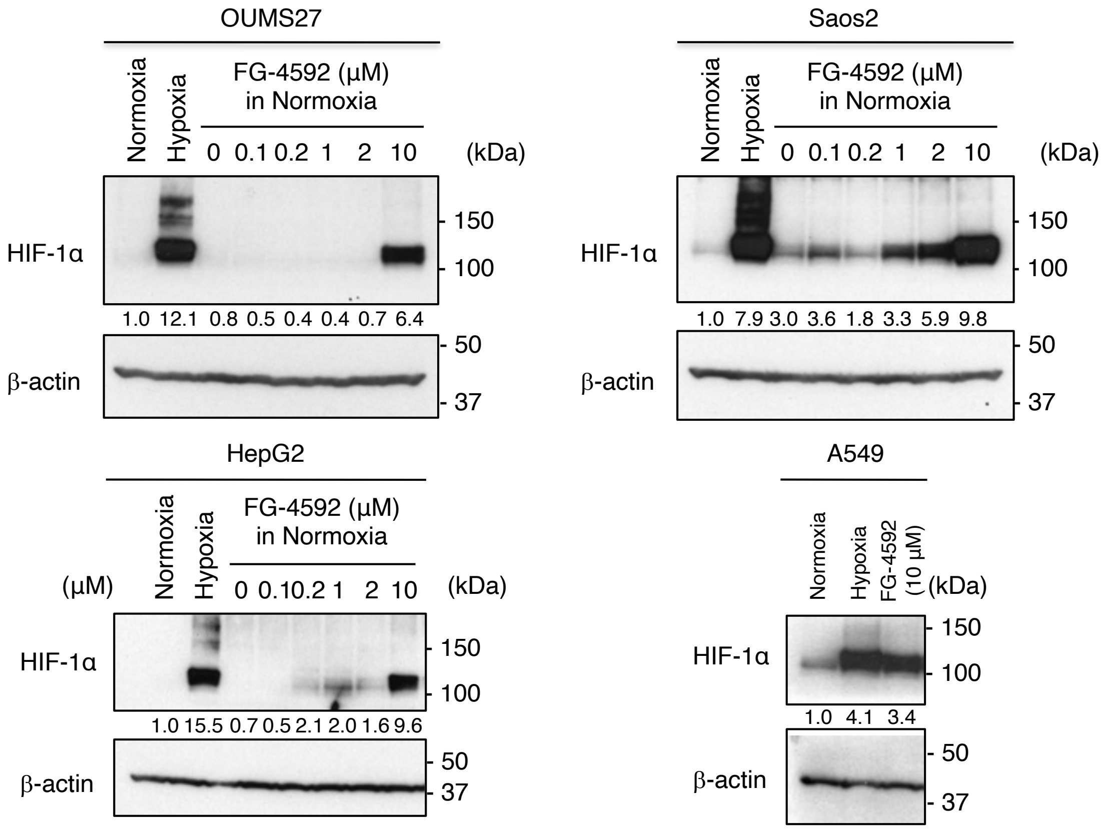

Figure S1. Protein levels of HIF-1α in OUMS27 (chondrosarcoma), Saos2 (osteosarcoma), HepG2 (hepatoblastoma), and A549 (lung adenocarcinoma) cells treated with normoxia, hypoxia, or various concentrations of FG-4592 were analyzed by immunoblotting. β-actin was used as an internal loading control. Relative expression levels of HIF-1α protein were calculated with β-actin expression as the denominator for each sample, and the values are shown between panels.

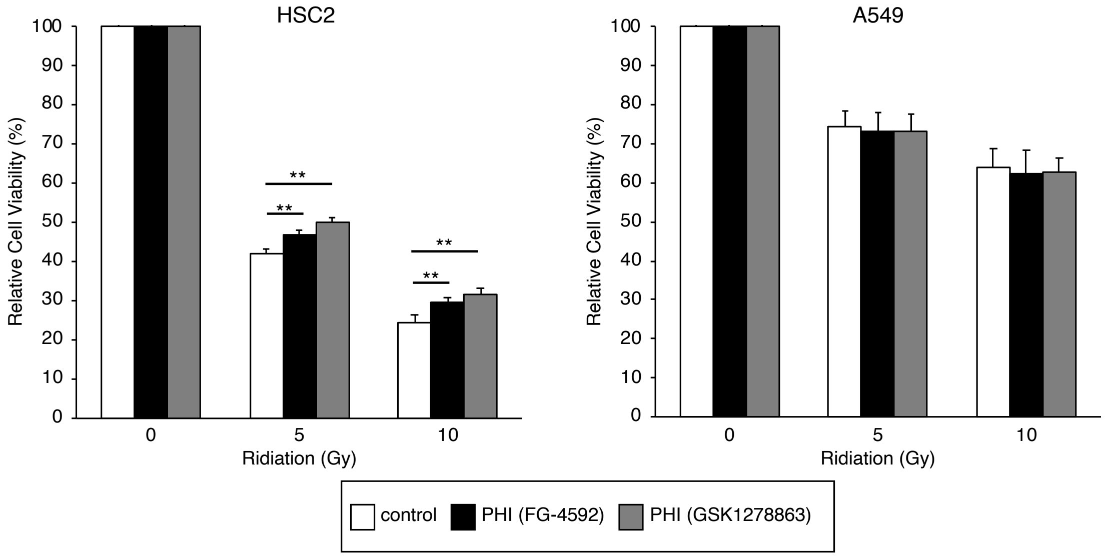

Figure S2. Effects of PHI treatment on radiation-induced cell growth inhibition. Relative cell viability of HSC2 (left) and A549 (right) cells after γ-irradiation (0, 5, or 10 Gy) under the indicated treatments with PHIs (10 μM FG-4592 or 10 μM GSK1278863) was evaluated by MTT assay. Data are presented as mean and SD ($n = 6$); $^{**}p < 0.01$ (for comparison with control).

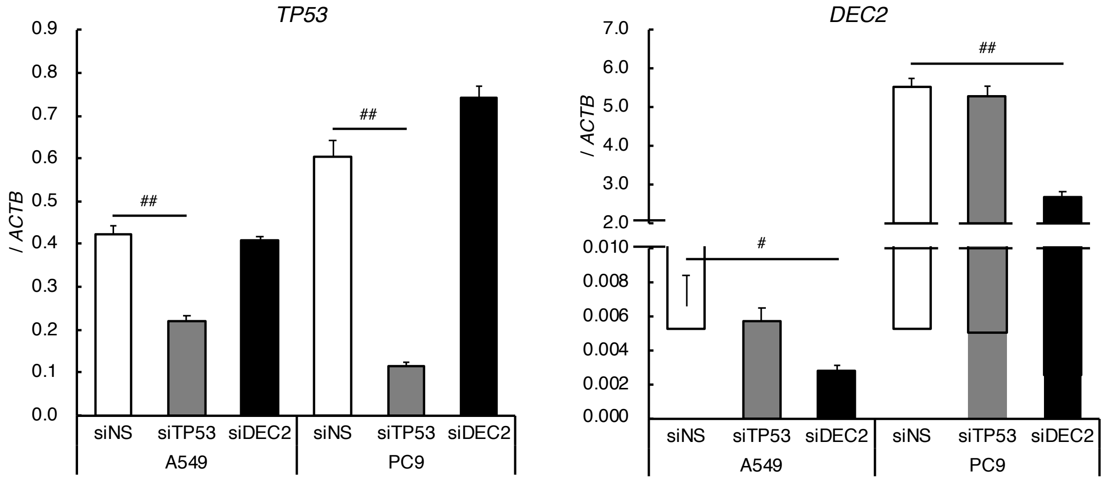

Figure S3. Effects of specific siRNA for *TP53* or *DEC2* in A549 and PC9 cells. Expression of *TP53* and *DEC2* in siNS (non-specific siRNA), siTP53 (siRNA for *TP53*), or siDEC2 (siRNA for *DEC2*) transfected A549 and PC9 cells were analyzed with quantitative RT-PCR. Relative gene expression levels were calculated as the ratio to that of *ACTB*. Data are presented as mean and SD (n = 3); #p < 0.05; ##p < 0.01 (for comparison between experimental samples).
